# Annotations

**Published:** 1909-10-16

**Authors:** 


### Annotations.

### Medical Men in Parliament.

The recent sudden death of Dr. G. J. Cooper, member of Parliament for Bermondsey, must reduce the number of medical members of the present House of Commons unless Dr. A. Salter, the Socialist candidate, should succeed in his candidature for the vacant seat. Dr. Salter is the senior member of a firm of four partners practising in Bermondsey. He is an old Guy's man, and his academical record has been a brilliant one, including a gold medal and first-class honours in three subjects at the London University M.B., two scholarships, and two exhibitions. Dr. Salter is, besides M.D. London, also D.P.H. and M.R.C.S., L.R.C.P., and he has done a good deal of original work in pathology and bacteriology. If returned, he should be of value in the House of Commons as an up-to-date and enlightened authority on matters medical, and a useful counter-check on the anti-vivisectionist tendencies of so many of those with whom he is politically associated.

### Food Reform.

The first annual report of the National Food Reform Association, which has just been issued, contains some remarkable evidence. When we consider that on an average 25 per cent. less food than is necessary for the maintenance of merely physical efficiency is being received by unskilled labourers, that trained artisans are receiving just about enough, and that members of the middle classes are receiving more than 15 per cent. over what is their due, it is at once apparent that there is need for better and more appropriate adjustment in the diet of the people. As one of the writers in the report aptly remarks, it is high time that the public should receive reliable instruction as to the value of food stuffs, and how to obtain proper nutrition with economy and with the least amount of household slavery. It is, indeed, a vital subject which urgently demands attention. Through ignorance there exists appalling waste which ought to be, and could be, easily avoided. We cordially commend the Association to the consideration of every one who is interested in food reform.

### The Alleged Dangers of Forced Feeding.

The rather abnormal hatred of control, in any form, associated with democracy (until, according to Plato and other sages democracy ends in tyranny) is doubtless the reason of the attitude of some people in regard to the treatment of wilfully starving "suffragists." Without plunging into the deep waters of philosophy, or the rather shallow ones of contemporary politics, or repeating what we have already stated in an editorial on the subject in a former issue, we may still enter an emphatic demur to Mr. Keir Hardie's statement: "the last man who died was treated in this way." The remark does not make it clear who exactly the last man to have a feeding-tube passed on him was, and to find this out would be nearly as difficult as to ascertain who at any given time was the last person to take a pinch of snuff. But if he died as a consequence of his meal, he must have been an uncommonly unlucky individual. In the hospitals of this country alone there must be very many patients who have come to look on the nasal tube, as the saying is, as their best friend; while in asylums, lunatics, who would otherwise quickly starve to death, are kept alive in this way for years. In view of all this, a reference made to "brutal methods of barbarism" hardly seems inspired by the common sense which should mark the practical legislator. A medical member of Parliament, who ought to know, has instanced the founding of the Claybury laboratory as a proof that democratic bodies are sympathetic towards medico-scientific ideas. In spite of this excellent authority, the manner of Mr. Keir Hardie's heckling of the Home Secretary does not suggest as much.

### Boric Acid in Cream.

That general toxic effects are produced by large or constant doses of boric acid is a fact not generally known, although in practice the toxicity of boracic acid solutions is well recognised. There has, however, been a decided difference of opinion as to the relative toxicity of minute quantities of the salts of boron when taken into the system in the form usually employed by those who contend that they are legitimate and harmless food preservatives. In some cases signs of irritation of the stomach and intestines have undoubtedly followed the ingestion of such minute doses, but it must be borne in mind that these cases can be explained on the supposition that certain persons possess a well marked idiosyncratic sensitiveness to boron salts. It is still, therefore, more or less an open question to what extent the use of these salts as food preservatives is permissible. Recently Dr. J. M. Hamill has investigated some aspects of the matter, and has presented a report on his researches to the Local Government Board. He concludes, after an elaborate and interesting survey of the whole case for and against boric acid preservatives, that a minute quantity of boric acid, borax, or mixtures of boron salts is harmless, and may safely be added to cream. He recommends the fixing of a maximum limit of boron preservative calculated as boric acid, and the enforcement of a rule to the effect that the presence of such preservative should in every case be declared. Such "declared cream" should be strictly differentiated from the pure produce which has had no preservative added. In Germany, America, France, and, indeed, generally abroad, the use of boron preservatives is not permitted, and it is undesirable that any change should be made in this country in existing regulations. Dr. Hamill's conclusions are interesting, but he has by no means wholly effaced the case against the use of such preservatives, and his recommendations seem to us to be open to grave objections.

